# Assessing Children’s Time-Use in Relation to Physical Fitness and Risks of Obesity and Diabetes: Development of a New Physical Activity Self-Report Instrument

**Published:** 2015-08-10

**Authors:** Y Cui, J Guo, M Santiago-Torres, D Schoeller, S Esmond, D Allen, M Henderson, A Rendon, A Carrel

**Affiliations:** 1 Civil and Environmental Engineering, University of Wisconsin-Madison, Madison, WI; 2 Nutritional Sciences, University of Wisconsin-Madison, Madison, WI; 3 Institute for Clinical and Translational Research, University of Wisconsin-Madison, Madison, WI; 4 Department of Pediatrics, University of Wisconsin-Madison, Madison, WI; 5 United Community Center, Milwaukee, WI

**Keywords:** Reliability, Validity, Activity Patterns, Metabolic Health

## Abstract

**Background:**

This study introduces a novel self-report instrument to measure children’s time-use in physical and sedentary activities and examines the relationships between children’s time-use and physical fitness and risks of obesity and diabetes.

**Methods:**

The new instrument utilizes a series of timelines, each representing an activity type. 188 children (53% girls) aged 10 to 14 year-old participated in the study. Their time-use data for two weekdays and one weekend day were collected. Anthropometrics and cardiovascular fitness were measured and children’s BMI z-score and PACER z-score were computed. One-time blood draw for fasting glucose and insulin were used to calculate insulin resistance using homeostasis model assessment for insulin resistance (HOMA_IR_).

**Results:**

The reliability assessment of this instrument indicated a moderately reproducible procedure (ICC > 0.6) for six activity types. The validity correlation for motorized travel time was high (r = 0.226, *P* < 0.05) between self-report instrument and GPS tracks. PACER z-score was positively correlated with time-uses of play (r = 0.159, *P* < 0.05), and organized sports (r = 0.198, *P* < 0.05); and was highly inversely correlated with BMI z-score (r = −0.441, *P* < 0.0001) and HOMA_IR_ (r = −0.472, *P* < 0.0001). Overall, only 14% of the children had physical activity for more than 60 minutes daily over three observation days.

**Conclusions:**

This instrument is particularly useful in assessing children’s activity patterns, especially for specific physical activities. The new instrument provides a reproducible measure of children’s perception of their activities. Our results emphasize the temporal context which is critical to formulating effective interventions targeting physical activity increase in children. Further efforts are needed to understand the differences between activity time obtained by the new self-report instrument and GPS tracks.

Although rates of pediatric obesity has stabilized in some countries, rates in many countries continue to increase. Moreover, various country wide efforts to reverse these secular trends have not yet been successful. Public Health professions have thus continued to develop and implement interventions toward reducing obesity rates. To this end, there is a continued need for methods to assess change in physical activity. We have developed a graphic assessment tool for use by children 10 years of age and over. This tool showed good reproducibility as well as some correlation with an objective GPS based measured of some activities.

Although the rate of increase has abated, obesity prevalence among U.S. young population aged 2–19 years remains at about 17% [1]. Obese children have an increased risk of insulin resistance, which can negatively influence glucose metabolism and increase risk of type 2 diabetes (T2D) [2]. Past research has demonstrated the association between childhood obesity and a broad spectrum of factors at the individual, family, and community levels [3]. In addition to diet, one factor that most researchers agree upon as being a contributor to childhood obesity and in particular related to metabolic disease is the physical inactivity [4, 5]. The U.S. Department of Health and Human Services recommends that children and adolescents should spend at least 60 minutes daily on moderate-to-vigorous physical activity to maintain a healthy life [5]. Regular physical activity is believed to increase physical cardiovascular fitness in children [6], counteract the negative effect of obesity on insulin resistance [7], and help lower the risk of conditions such as obesity and T2D [8], whereas sedentary behavior is associated with increased risk of metabolic disease in children [9, 10].

Interest in research on how children organize their day is growing because of the increasing need for effective evaluations of interventions to promote physical activity participation and further prevent obesity and T2D in children. This calls for accurate, reliable and feasible measurement instruments that provide deeper insight into the nature and extent of children’s physical activity patterns and time-use.

Past studies have used epidemiological data collected by activity recall instruments to show the influence of time-use and physical activity on children’s risk of metabolic disease, including insulin sensitivity [11], cardiovascular fitness [12], and weight status [8, 13, 14]. Some of these studies collected physical activity data using objective measures such as motion sensors, accelerometers, heart rate monitoring, doubly-labeled water and GPS recording [12, 14]. Although these measurements require low administration and respondent burden, they are unable to capture sedentary activities or provide data on the context at times. Other studies used self-report instruments to assess children’s physical activity participation, including activity questionnaires, activity diaries, activity logs, and activity recalls [15]. The self-report instruments are relatively inexpensive and easy to administrate compared to objective measures.

Basically, there are two main approaches used in children’s self-report instrument: activity-based and time-based [16]. The Self-Administered Physical Activity Checklist (SAPAC) [17], the Seven-Day Physical Activity Recall (7DPAR) [18], the Four by One-Day Recall [19], and the Typical Day Recall [20] are activity-based instruments. These tools examine a participant’s time-use within a conceptual time-frame by asking him/her to report his/her time spent in specific activities on certain day(s) within a week. The activity-based instruments are usually structured around a list of activities reflecting different intensity levels, which are convenient to calculate energy expenditures [21]. With little cue about the time of day, these instruments are unlikely to elicit information on activity frequency and time-of-day occurrence. Unlike the activity-based approach, the time-based approach specifies bounded time periods, dividing a recalled day into 15-min or 30-min segments in a time queue. The Physical Previous Day Physical Activity Recall (PDPAR) [22] and the Three-Day Physical Activity Recall (3DPAR) [23] use time-based approach. These self-reports require a participant to select a dominant activity for each time block from a list of activities which are grouped into several categories, such as sleep/bathing, eating, transportation, and physical activities/sports. When reporting the start and end time of the designated activity, the participant also needs to rate activity intensity. This type of instrument is advantageous in collecting data on the frequency, intensity, and time-of-day of a wide range of physical activities.

The aim of this study was to introduce and examine the reliability and validity of a new instrument – the Graphs for Recalling Activity Time (GReAT), an innovative physical activity self-report recall instrument developed for school-aged children. Secondly, children’s cardiovascular fitness, body mass index (BMI) and insulin resistance were measured and examined in relation to children’s physical activity. Finally, the new instrument was compared against objectively measured time in active and inactive pursuits. In general this study set out to address the limitations of other physical activity recall instruments for children, and to provide new evidence on the relationships of time-use of different activities and children’s physical fitness and risks of obesity and T2D.

## Methods

### Study Background

The present study was nested within a large-scale multidisciplinary research project, Healthy Activities Partnership Program for Youth (HAPPY), a community-engaged research project examining factors that impact Hispanic children’s health in the built, nutrition and social environments surrounding a charter school in Milwaukee, Wisconsin [24, 25, 26]. Before data collection, the University of Wisconsin-Madison (UW-Madison) Institutional Review Board (IRB) approved this study. Parental consent forms and student assent forms were obtained from each participant. The study protocol was implemented in the school as part of the school curriculum. A 100% retention rate of those consented and assented participants was achieved and maintained throughout the project.

### Participants

The study target population was the students attending the Bruce Guadalupe Community School (BGCS) at the United Community Center (UCC) in Milwaukee, WI. The UCC is a social service agency serving Hispanic families with programs ranging from pre-k school education to senior programs. Most of the students (98%) who enrolled in K-3 to 8th grades at the BGCS are Hispanics. About 45% of the families preferred to speak Spanish at home, 43% preferred both Spanish and English, while only 12% preferred English. 66% of the families reported annual incomes of $35,000 or less.

Successful recruitment strategies included using UCC endorsed introductory study materials to the students’ homes, and providing study information during well attended family events at the school. Inclusion criteria of study population were applied, including academic attendance at the BGCS from 5th grade to 8th grade, cognitive ability to understand instructions for research-related activities, and compliance with data collection methods.

A total of 188 students (53% girls) and 173 of their parents were enrolled in this study. The ethnic composition of students was determined by the parental-reported Demographic Survey, with 99% of them are Hispanics. There were 67% of students who were between 12 and 14 years of age; 31% of students were younger than 12 and 2% were older than 14.

### Instrument

The GReAT was developed using a timeline chart to report previous day’s activities. Specifically, each timeline allows recording the engagement in an activity type with the name of that activity type listed above the timeline. The name of the activity type is followed by a brief question asking whether the participant did the activities of that activity type on the previous day. Each timeline represents a day starting from 5:00 a.m. to 12: 00 a.m. with each hour divided into four 15-min intervals. Due to children’s limited understanding of the timeline chart concept, three time-of-day sections (i.e. Morning, Day, and Evening/Night) are marked along the timeline in order to visualize how a day is composed. Additionally, every time stamp is labeled along the timeline every hour.

Utilizing a series of timeline charts, the GReAT assesses 14 activity types - Napping, Study, Active travel (e.g. walking and cycling), Non-Active Travel, Watch TV, Play Video Game or Computer Game, Other Non-Homework Related Computer Use, Phone Use, Sport, Play, Meals (i.e. Breakfast, Lunch, and Dinner), and Eat Snacks. Time spent in sleep is assessed in text only. For each timeline, the participant is first required to check whether he or she did the activity within the scope of that activity type on the previous day. If the participant did participate in that activity type, he or she should check “Yes” and then recall the start and end time by blackening the corresponding time slots for each activity episode on that timeline. Otherwise, this participant should check “No” for that timeline.

For the Meals, the participant is asked to indicate where he or she had that meal (at home, at school, or at restaurant). If the participant had that meal at a restaurant, he or she also needs to report the name of the restaurant. Furthermore, questions on nutritional intake are asked along with each meal timeline, asking the participant to mark all food items that he or she had during that meal on the previous day. A complete GReAT form can be requested from the corresponding author.

## Procedures

The data collection occurred between Fall 2010 to Spring 2012 with 188 students, assessing one class of students per week. During a given week, on the 1st day (usually a Friday), the research staff provided a 20-min training session to the students first and then let them practice filling out some activity timelines in the GReAT form. Through this process, students became familiar with the self-report instrument and could have questions answered by project staff. During this session, the GPS (global positioning system) units were assigned to each of the students and they were required to wear it for a week (with the exception of sleeping and showering) to track their movement, including total distance moved and time of movement [25]. On 2nd, 3rd, and 4th days (usually Monday, Wednesday or Thursday, and Friday) of the following week, physical activity data were collected on each of the three occasions using GReAT under administration of the UCC staff. On the 4th day, the UCC staff took the GPS units from the students and downloaded the GPS data to computer.

Data on students’ cardiovascular fitness, BMI, and insulin resistance were separately collected. During the 2010–2011 academic year, their weight was measured using a beam balance and height was measured using a stadiometer in physical education class at school with shoes off and light clothing. Their BMI was calculated as weight in kilograms divided by height in meters squared (kg/m^2^). The cardiovascular fitness was assessed using the Progressive Aerobic Cardiovascular Endurance Run (PACER) test, a multistage 20-meter shuttle run administered at school by research staff. During this test, a participant ran back and forth along a 20-meter track, and each minute the pace to run the track increased. The initial running speed was 8.5 km/hour, and increased by 0.5 km/hour every minute. The test finished when the participant failed twice to complete the 20-meter run in the allotted time. As the number of laps per minute increased with the running speed, the PACER result was represented as the number of laps successfully completed.

For assessment of insulin resistance, a separate assent and consent were obtained from 101 of the 188 students to provide a fasting blood draw. For each participant, a phlebotomist obtained 5–10 ml of blood from him or her on a school day morning. These samples were transported to the UW-Madison Hospital and Clinics on ice for processing. Insulin resistance was assessed using a single blood test of fasting glucose and insulin levels, referred as derived homeostasis model assessment (HOMA_IR_).

### Data Processing

Only numerical data collected by GReAT were considered in this study. Of the 188 student participants, 163 of them completed the 1st self-report, 171 of them completed the 2nd self-report and 172 of them completed the 3rd self-report. Physical activity data were coded electronically, recording the start and end time (e.g. 15:00 p.m. and 15:45 p.m.) for all activity episodes the participants had undertaken. In order to acquire the time-use of each activity type, start time was subtracted from end time to derive the duration for each activity episode of that activity type. The durations of all activity episodes for that activity were added up to obtain the total time spent in that activity type.

The PACER test results were calculated as standard deviation score based on data of 27,000 WI students grouped by age and gender, represented as PACER z-Score. A z-score of 0 is equivalent to the median or 50% percentile value, a z-score of 1.00 and more is approximately equivalent to 84% percentile, a z-score of 2.00 and more is approximately equivalent to 98% percentile and a z-score of 2.85 and more is equivalent to 99% percentile. Three categories of PACER percentiles were defined in accordance with Carrel and colleagues’ study [27].

### Test-retest Reliability and Validity of the GReAT

The reliability assessment of this new physical activity self-report instrument was based on two self-report recalls made six hours apart on the same data collection day among 15 students (mean age and standard deviation = 13.3 ± 0.46). Their time-use of each activity type was calculated in order to determine the reliability of the instrument. The intra-class correlation coefficient (ICC) for continuous variable has been used as a measure of test-retest reliability [28]. The ICC value that is greater than 0.60 is considered acceptable [29].

The validity of the new instrument was determined using the matched GPS tracks data [25]. The total number of minutes of self-reported activities via GReAT was directly compared to the number of minutes for similar intensities of activity derived from the GPS tracks data. The Pearson’s correlations were computed for the 3-d (of GReAT data collection) as a whole.

### Data Analysis

Descriptive analysis was conducted to verify sample absence-of-bias and variable normality. HOMA_IR_ was logarithmically transformed to normalize its distribution. In particular, the average daily time-use of each activity type was obtained from students who completed 1st self-report and at least one of the 2nd and 3rd self-reports. A few students were absent on certain data collection day(s). 29 activity episodes were inaccurately recalled based on research staff’s observations. As a result, invalid self-report data were excluded from data analyses.

Analysis of variance (ANOVA) was used to examine gender differences in cardiovascular fitness, HOMA_IR_, and time-use of different activity types. It was hypothesized that the amount of activity time that GReAT accounted for decreased with successive self-reporting among participants. Multiple linear regression analysis was conducted to examine the independent associations between total self-reported activity time and reporting number. Pearson’s correlation test was applied to examine the bivariate relationships between physiologic characteristics and time-use of different activity types. A series of explanatory analyses were performed to explore students’ activity patterns, focusing on their engagement in doing sports and screening activity. All statistical analyses were performed in the Statistical Package for Social Sciences (SPSS version 20, Chicago, IL).

## Results

### Descriptive Statistics

A total of 188 students formed the final sample for data analyses. Salient sample characteristics were summarized, including gender differences in age, BMI z-score, PACER z-score, and HOMA_IR_, see Table 1. Analysis revealed that PACER z-score (*P* < 0.001) and time-use of Video-game Playing (*P* < 0.05) were significantly higher in the male students versus female students. There were no other significant differences between the students by gender.

### Test-retest Reliability

The test-retest correlations ranged from 0.060 to 0.977. The reliability assessment of this instrument indicated a moderately reproducible procedure (r > 0.6) for six activity types, including Sleep (r = 0.913, *P* < 0.001), Study (r = 0.706, *P* < 0.05), Active Travel (r = 0.801, *P* < 0.001), Non-Active Travel (r = 0.977, *P* < 0.001), Watch TV (r = 0.857, *P* < 0.001), and Sport (r = 0.950, *P* < 0.05).

### Validity

Table 2 presented the correlations between the total number of minutes of reported activities and minutes recorded by GPS units. The total number of minutes of self-reported physical activity was computed by adding up minutes reported for Sports and Play. For self-reported sedentary activity, its total number of minutes was computed by adding up minutes reported for Watch TV, Computer Use, and Play Video Game. The overall correlations between the new self-report instrument and objective GPS measure were weak for physical activity and sedentary activity. The overall correlation for motorized travel was significant and for 6th graders the validity correlation was higher.

### Reporting Fatigue

For the entire cohort, there was evidence of a small reporting fatigue. Total minutes of day reported, which includes wake and sleep periods, decreased about 4% with each successive day (Table 3). The beta coefficients from multiple linear regressions implied that this was largely driven by the 5th grade students, who were actually accounting for more minutes for the first report than there are minutes in a day. As such, we cannot determine if this is reporting fatigue or a more accurate report. For 6th, 7th and 8th graders, the trend between successive reports was not significant.

### Pearson’s Correlations

For the physical activity, PACER z-score was correlated with Active Travel (r = 0.179, *P* < 0.05), Sport (r = 0.198, *P* < 0.05), and Play (r = 0.159, *P* < 0.05). When looking at the correlations between physiologic characteristics, PACER z-score was highly inversely correlated with BMI z-score (r = −0.441, *P* < 0.0001) and HOMA_IR_ (r = −0.472, *P* < 0.0001). BMI z-score was also correlated with HOMA_IR_ (r = 0.705, *P* < 0.0001).

### Exploratory Analysis

**Fig: 1:**
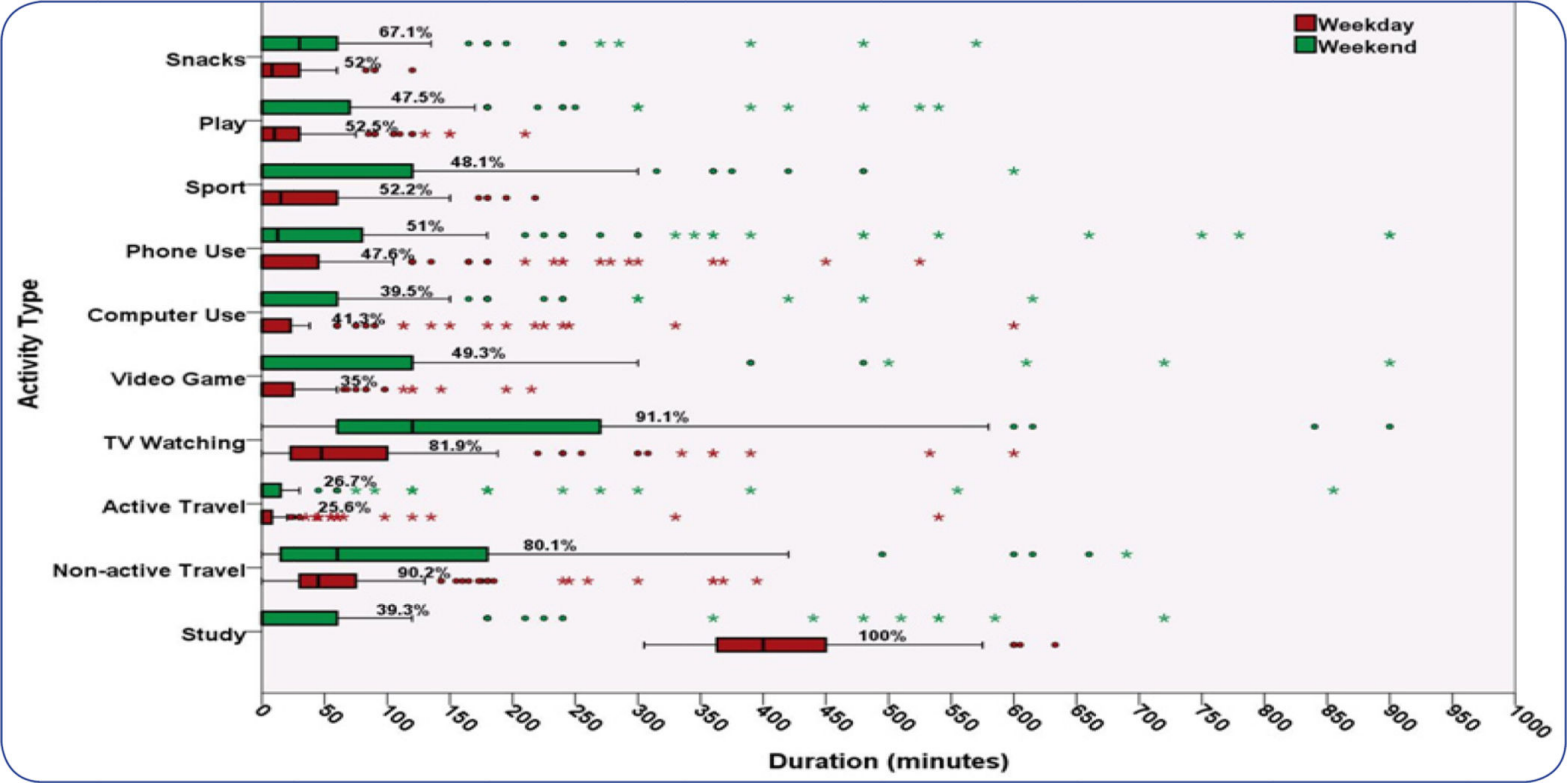


**Fig: 2:**
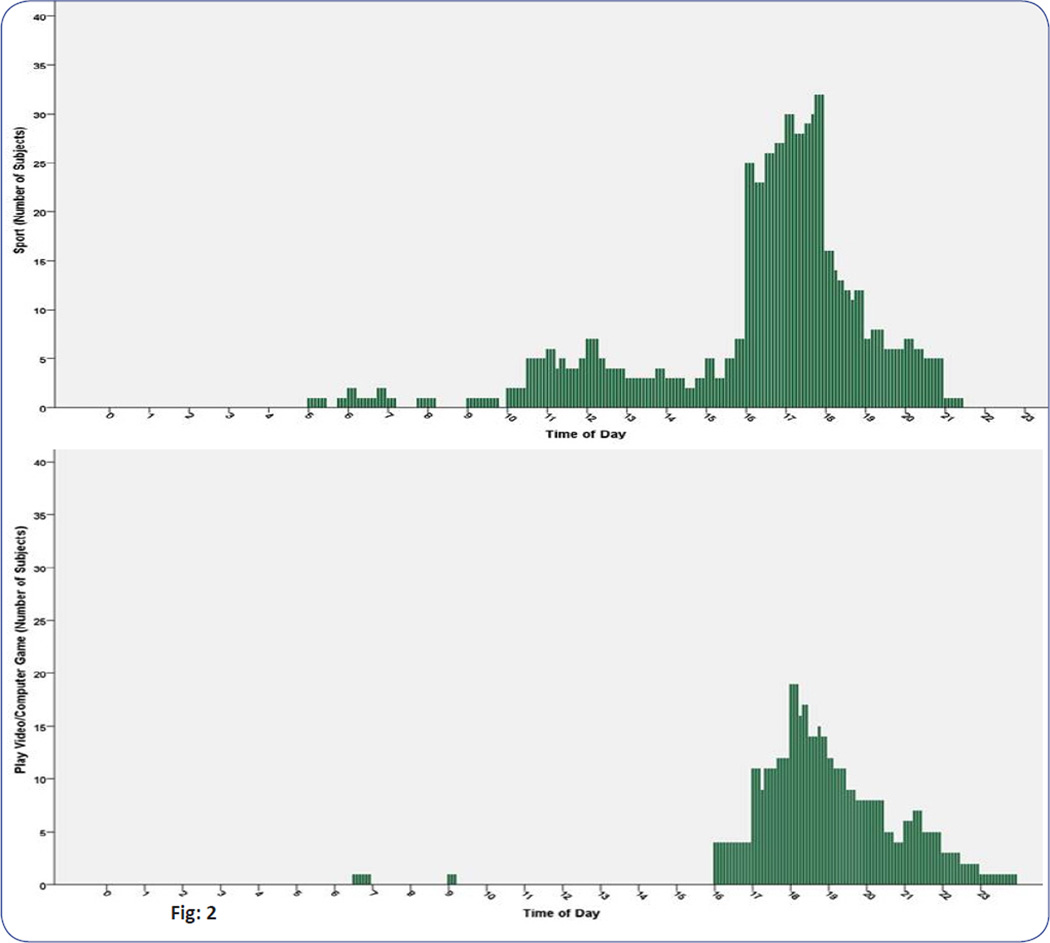


Figure 1 depicted substantial variations in students’ time-use patterns between weekdays and weekend days, particularly for the discretional activities. On weekend days, the mean durations of TV Watching (118 minutesD^−1^) was the highest among all activities assessed, with 91% of the students in the study reported Watching TV on weekend days. Participation rate of Watching TV was According to the Pearson’s correlation test, only physical fitness was significantly correlated with several time-uses of different activity types. For the sedentary activity, time-use of Phone Use was inversely correlated with PACER z-score (r = −0.199, *P* < 0.05). second only to Study on weekdays. Additionally, if focusing on the out-of-school time only, it is surprising to note that there were a great amount of students engaging in TV Watching from afternoon till evening, as shown in Figure 2.

To examine whether the students in this study met the recommendation of at least 60 minutes of moderate-to-vigorous physical activity daily, we investigated their physical activity engagement at different levels, as shown in

## Discussion

The current study introduced a novel physical activity self-report instrument (GReAT) and established the reliability of this timeline-based instrument. The GReAT was uniquely designed for school-aged children and overcame some of the inflexibility related to existing self-report physical activity instruments. For example, existing measurement instruments usually collect children’s physical activity data as average hours per week [30] average minutes per day [14, 31] number of times per week [8, 32] energy expenditure per day [12] or activity levels [13]. The GReAT is advantageous in its flexibility of providing summary information (total time-use and frequency) across a specified period of time (from 5:00 a.m. to 12:00 a.m.) for specific physical activities and sedentary activities that can be represented in any of these formats. The GReAT assessed 1-d snapshot of children’s perceived activities on three occasions, including one weekend day and two weekdays, to get a more complete picture of their physical activity patterns.

In the study, the test-retest reliability coefficients indicate that children’s recall of their previous day’s time-use of Sleep, Study, Active Travel, Non-Active Travel, Watch TV, and Sport was stable when assessed twice on the same day with six hours apart. The low reliability results of Meals (r = 0.413, *P* > 0.05) and Play (r = 0.060, *P* > 0.1) in the current study could be attributed to the sporadic nature of these activities. Similar finding can be located in another study [33], where the authors found that reliability results for organized activities were higher than non-organized activities.

The validity correlations between the GReAT and objective measure were low and inconsistent for physical activity and sedentary activity. Even though the results indicated limited validity of physical and sedentary activity self-reports, the significant correlations for motorized travel were still valuable in verifying GReAT’s validity in reporting children’s travel patterns. Furthermore, the GPS units advantage in its capability of recording movement tracks and movement’s geographic location. Compared to other activities with possibly multiple locations to take place, the motorized travel was easier to identify and derive from the GPS tracks data.

The most salient three findings are discussed next. First, it is important to point out that we did not find a relationship between children’s reported activity engagement and measure of obesity risk (BMI z-score) or diabetes risk (HOMA_IR_). There may be other environmental modifiable risk factors for obesity and diabetes risks that were not included in this analysis. And these factors can be dietary habits and genetic predisposition to obesity and diabetes risks. As reported elsewhere, we did find a significant relationship between children’s risk of obesity and dietary behaviors, indicating that diet had a stronger influence on BMI than physical activity [26] In contrast to the relationships with BMI z-score and HOMA_IR_, we found significant relationships between children’s reported physical activity engagement and cardiovascular fitness (PACER z-score). The positive correlations suggest that longer time-uses of organized sports, active travel, and play were associated with higher physical fitness. These findings point out the importance of understanding the temporal context of how children organize these physical activities in their day, for effective formulation of interventions.

Second, active travel has been recognized as an opportunity to improve physical fitness and reduce obesity risk [32]. In this study, children’s participation rates in active travel were relatively low on both weekend days (27%) and weekdays (26%). Actually, our research staff learned that approximately 80% of the children attending the BGCS lived within a mile from the school. We discussed the results with UCC staff and learned that despite the close proximity, there were several unsafe intersections to cross. In fact, many parents worked shifts such that they needed to safely drop children off at school before/after a work shift. It presents a challenge to establishing and sustaining physical activities outside of the school setting, given realities of these families’ lives.

Third, from the time-of-day distribution of Sport and Play Video/Computer Game on a school day, it can be observed that a significant portion of children were engaged in doing sports after school hours, from 4pm into the evening (Figure 2). This reinforces the important role the school/community provided in terms of a safe and suitable environment for local sporting activities. This observation prompts the question of whether, and how, we might be able to help those children, who played computer and video games during after school and evening hours, to spend their time in sports instead. Furthermore, there were only a small number of children (14%) who met the national guidelines which recommends that children should engage in physical activity for more than 60 minutes daily. Yet, this rate is much lower than the national rate in the 2011 Youth Risk Behavior Survey, which showed that, nationwide, 49.5% of children aged nine to thirteen years had been physically active at least 60 minutes per day on five or more days during the week for which the data was collected [34]. It can be concluded that school interventions are in need to promote physical activity among children who did not undertake enough physical activity after school or in the weekend.

The current study is subject to several limitations. First, the homogeneity of participants (99% Hispanics) prevented us from generalizing the results to other population groups. Second, the new self-report instrument was not validated against other measures for most of the individual items. We did, however, compare active and sedentary summary variables against GPS track data and except for motorized travel; comparisons were disappointingly weak which may be a general limitation of self-report. Other objective measures such as heart rate monitor, can be introduced to provide validity test of the GReAT to increase its value. Third, we did not control for the familiar and environmental factors, which may account for the unrepresented relationships between risks for obesity and T2D and time-use. Fourth, the use of cross-sectional data collected was not sufficient to interpret cause and effect in the relationships between time-use of physical activities and physical fitness.

Future research should be directed toward implementing GReAT in a diverse group of children. Further efforts are needed to understand the differences between activity time obtained by the new self-report instrument and GPS tracks. Current recall time-frame can be expanded to a longer term to derive children’s habitual activity patterns and evaluate its impact on their metabolic risks. Longitudinal designs should be conducted to investigate the causality of any relationships between physical activity and physiologic factors.

## Figures and Tables

**Table 1:** 

	All	Boys	Girls
Sample Size	188	89	99
Age (years)	11.90 ± 1.37	11.99 ± 1.43	11.82 ± 1.33
BMI z-score	0.94 ± 0.94	1.01 ±0.98	0.88 ± 0.91
PACER z-score	0.36 ± 0.93	0.63 ± 0.97	0.11 ± 0.84**
**PACER percentile categories**^a^,^b^			
Low fitness: < 33%	25 (15%)	6 (8%)	19 (22%)
Moderate fitness: ≥ 33% to <67%	69 (42%)	27 (34%)	42 (48%)
High fitness: ≥ 67%	72 (43%)	46 (58%)	26 (30%)
HOMA_IR_^c^	0.54 ± 0.24	0.51 ± 0.28	0.56 ± 0.21

a ANOVA was not applied to PACER percentile categories.

b Source: Carrel AL et al. *J Pediatr* 2012;161:120–4.

c the sample size of HOMA_IR_ is 100 (56 girls).

** *P* < 0.001 and *P < 0.05 for significant gender differences.

**Table 2:** 

	All Participants	5th Graders	6th Graders	7th Graders	8th Graders
Motorized Travel	0.226* (100)^a^	0.062 (20)	0.580** (20)	0.114 (35)	−0.111 (25)
Physical Activity	−0.133 (25)	−0.043 (4)	−0.593 (4)	−0.179 (8)	0.111 (9)
Sedentary Activity	−0.063 (152)	0.158 (23)	−0.101 (28)	−0.285* (56)	−0.172 (45)

a number of observations is presented in parenthesis.

* *P* < 0.05;

** *P* < 0.01;

*** *P* < 0.001.

**Table 3:** 

	Dependent Variable: Total Reported Activity Time				
	All Participants^b^ (N = 141)	5th Graders (N= 42)	6th Graders (N= 36)	7th Graders (N= 32)	8th Graders (N= 31)
Constant	1421.773***	1626.667***	1372.056***	1310.688***	1316.581***
2nd Report^a^	−46.652	−205.952*	36.750	6.250	17.710
3rd Report^a^	−114.837*	−288.452**	−111.361	−77.094	77.387

a dummy variable.

b Participants who completed three activity recalls.

* *P* < 0.05;

** *P* < 0.01;

*** *P* < 0.001.

**Table 4:** Number of Participants Engaged in Different Levels of Physical Activity (PA)

Classification^a^	All	Boys	Girls
Sample size^b^	135	63	72
Engaged in PA > 60 min for 3 days	19 (14%)	12 (19%)	7 (9%)
Engaged in PA > 60 min for 1 or 2 day(s)	83 (62%)	35 (56%)	48 (67%)
Not Engaged in PA > 60 min for 3 days	33 (24%)	16 (25%)	17 (24%)

a physical activity includes active travel, organized sport, and active play.

b based on number of participants who correctly reported physical activity on each of three observation days.
